# 

**Published:** 1866-08

**Authors:** 


CONTENTS FOR AUGUST.

ORIGINAL CONTRIBUTIONS.

The Trichina Spiralis. A Family Poisoned by Eating Trichinous Pork. By E. M. Smith, M.D....... p. 337

Diagnosis and Treatment of Pott's Disease of the Spine. By F. O. Earle, M.D....... 342

Epidemic Cholera: Its Pathology and Treatment. By R. Dexter, M.D. 351

A Case of Dry Gangrene of the Leg, caused by Obstruction of the Common Femoral Artery—Amputation at the Middle Third of the Thigh—Recovery. By J. W. Freer, M. D....... 354

Surgical Cases. By Geo. K. Amerman, M. D....... 356

HOSPITAL REPORTS.

Cook County Hospital Surgical Clinics. By R. G. Bogue, M. D....... 360

BOSTON CORRESPONDENCE.

The American Ophthalmological Society....... 366

PROCEEDINGS OF MEDICAL SOCIETIES.

Chicago Medical Society....... 369

Rock River Union Medical Society....... 373

Morgan County Medical Society....... 374

Military Tract Medical Association....... 375

SELECTED ARTICLES....... 376

EDITORIAL.

Book Notices, etc....... 379

